# Mouse Microglial Calcitonin Receptor Knockout Impairs Hypothalamic Amylin Neuronal pSTAT3 Signaling but Lacks Major Metabolic Consequences

**DOI:** 10.3390/metabo12010051

**Published:** 2022-01-08

**Authors:** Bernd Coester, Thomas A. Lutz, Christelle Le Foll

**Affiliations:** Institute of Veterinary Physiology, Vetsuisse Faculty, University of Zurich (UZH), 8057 Zurich, Switzerland; bernd.coester@sund.ku.dk (B.C.); thomas.lutz@uzh.ch (T.A.L.)

**Keywords:** amylin, leptin, hypothalamus, AgRP, CTR, CALCR, microglia, IL-6, energy expenditure, adiposity, glucose tolerance

## Abstract

Amylin and leptin synergistically interact in the arcuate nucleus of the hypothalamus (ARC) to control energy homeostasis. Our previous rodent studies suggested that amylin-induced interleukin-6 release from hypothalamic microglia may modulate leptin signaling in agouti-related peptide expressing neurons. To confirm the physiological relevance of this finding, the calcitonin receptor (CTR) subunit of the amylin receptor was selectively depleted in microglia by crossing tamoxifen (Tx) inducible Cx3cr1-Cre^ERT2^ mice with CTR-floxed mice. Unexpectedly, male mice with CTR-depleted microglia (KO) gained the least amount of weight of all groups regardless of diet. However, after correcting for the tamoxifen effect, there was no significant difference for body weight, fat mass or lean mass between genotypes. No alteration in glucose tolerance or insulin release was detected. However, male KO mice had a reduced respiratory quotient suggesting a preference for fat as a fuel when fed a high fat diet. Importantly, amylin-induced pSTAT3 was decreased in the ARC of KO mice but this was not reflected in a reduced anorectic response. On the other hand, KO mice seemed to be less responsive to leptin’s anorectic effect while displaying similar ARC pSTAT3 as Tx-control mice. Together, these data suggest that microglial amylin signaling is not a major player in the control of energy homeostasis in mice.

## 1. Introduction

The co-secretion of amylin and insulin from pancreatic β-cells is part of the physiological response to a gastrointestinal influx of nutrients [1]. Amylin reaches the brain [2] and binds to its receptors mainly located on area postrema (AP) neurons [3,4,5], but also on hypothalamic neurons [6,7]. The amylin receptor is a heterodimer of the calcitonin receptor (CTR) and receptor activity-modifying proteins (RAMP 1, 2 or 3) [8,9]. In vivo studies using fluorescently labeled rat amylin have shown a strong signal in the AP, the hypothalamic arcuate nucleus (ARC), the median eminence, and the vascular organ of the lamina terminalis [2]. The presence of amylin receptor components in single ARC and AP neurons has been confirmed by studies using single cell qPCR, immunohistochemistry, and in situ mRNA hybridization [10,11,12,13].

In the ARC, amylin signaling in POMC neurons controls energy expenditure in adulthood [11,12,14] and during brain development plays a role in axonal fiber outgrowth of neurons projecting from the ARC to the paraventricular nucleus (PVN) [7,15,16] and from the AP to the nucleus of the solitary tract [17].

Many studies demonstrate that experimental weight loss treatments with amylin and leptin show additive and synergistic effects [18,19,20], possibly because of a “leptin-sensitizing” effect of amylin in the mediobasal hypothalamus [16,21]. Based on in vivo and primary cell culture studies, a working model for amylin and leptin interaction in the ARC was proposed whereby amylin binds to microglial CTR to stimulate interleukin-6 (IL-6) secretion, which enhances leptin-induced pSTAT3 signaling after binding neuronal IL-6/gp130 receptor complex [6,7,15,22]. Several lines of evidence support this hypothesis. First, amylin receptor components are present in dissociated rat primary microglia, astrocytes, and neurons of the VMH (ARC plus ventromedial nucleus (VMN)), but only rat VMH explants and primary cultured microglia respond to amylin exposure by producing IL-6. Second, blocking IL-6 signaling in vivo in rats decreases VMN leptin-induced pSTAT3 and exogenous amylin administration injection increases leptin-induced pSTAT3 in WT mice but not in IL-6 KO mice [15]. Cultured primary VMH neurons harvested from a leptin resistant, polygenic rat model of obesity treated with IL-6 for 5 days were found to restore leptin-sensitivity manifested primarily by increased expression of leptin receptor expression [23]. Finally, IL-6 KO mice have shown a decrease in AgRP but not in αMSH ARC to PVN axonal outgrowth [22].

Building on these results, we hypothesized that a genetic knockout of CTR in microglia would disrupt amylin-induced microglia IL-6 production resulting in reduced ARC leptin sensitivity. To this end, we used tamoxifen (Tx)-inducible Cx3Cr1-Cre^ERT2^ mice to selectively and temporally knock-out CTR in microglia and to assess the potential impact on energy balance and glycemic control in mice exposed to different diets.

## 2. Results

### 2.1. Cx3Cr1-Cre^ERT2^ Mice Show Specific Cre Activity in Microglia after Tamoxifen Treatment

**Figure 1:**
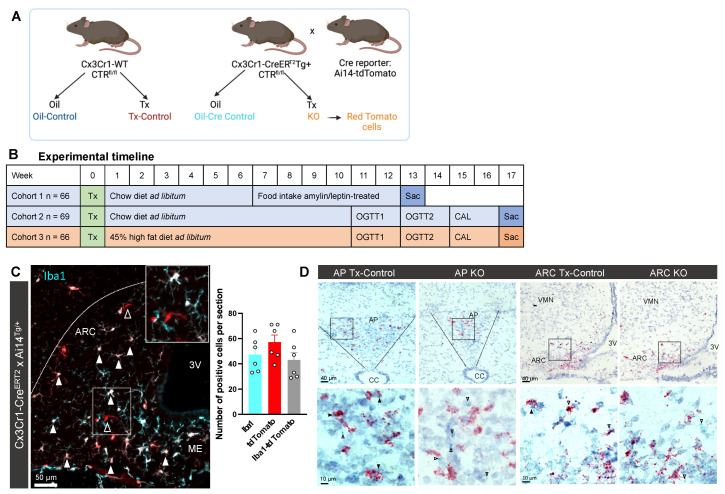
Specific expression of functional Cre recombinase after tamoxifen induction. (**A**) For metabolic phenotype studies, three cohorts of Cx3Cr1-Cre^ERT2^ mice were bred with CTR^fl/fl^ mice; (**B**) Mice were tamoxifen (Tx) induced at ≥56 day of age and fed ad libitum chow or 45% high-fat diet for several weeks before experiments. (**C**) Cx3Cr1-Cre^ERT2^ mice were crossed with an Ai14 tdTomato Cre-reporter line that expresses a STOP-floxed tdTomato allele. Immunohistochemistry with Iba1 antibody reveals a near-perfect overlap of Iba1 (cyan) with tdTomato (red) that results in a white color (filled arrowheads) with a few microglia only expressing tdTomato (empty arrowheads). (**D**) The model was further verified with Basescope in situ hybridization showing no CTR on microglia cells (empty arrowheads) in KO mice while Iba1-CTR colocalization was detected in Tx-control mice (filled arrowheads). ARC: Arcuate nucleus; ME: Median eminence; 3V: Third ventricle; CC: Central canal; AP: Area Postrema; OGTT: Oral glucose tolerance test; CAL: Indirect calorimetry, SAC: Sacrifice.

CTR^fl/fl^ × Cx3Cr1-Cre^ERT2^ mice crossed with an Ai14 TdTomato Cre-reporter line (Figure 1A) [24] produced a robust tomato fluorescent signal confirming the Cre activation after Tx treatment. The tomato signal overlapped with immunostaining of the ionized calcium-binding adaptor molecule 1 (Iba1, Figure 1C), a marker for microglia [25,26]; 91% of the Iba-1 positive cells were colocalized with tdTomato after Tx treatment. We then adapted this Tx protocol into the study design of the main experiments with five daily injections in adult mice as a starting point to observe their phenotype (Figure 1B). To further validate the model, we colocalized *Iba1* with *CTR* mRNA using in situ hybridization in the AP and ARC of WT, but not of KO mice (Figure 1D).

### 2.2. CTR Knockout in Microglia Has No Effect on Body Weight and Body Composition

**Figure 2:**
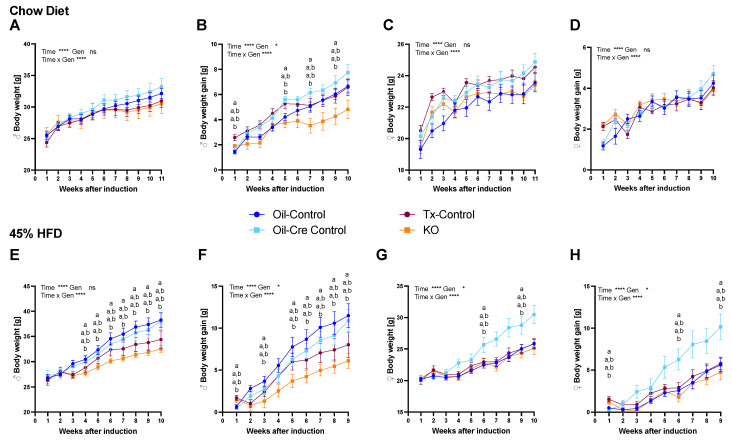
CTR depletion in microglia leads to lower body weight in chow- and HFD-fed male mice. Body weight on chow diet (cohort 2; (**A**,**C**)) and 45% HFD (cohort 3; (**E**,**G**)). Cumulative body weight gain after tamoxifen (Tx) or corn oil injection on chow (**B**,**D**) and 45% HFD (**F**,**H**). Values are displayed as mean ± SEM; n = 6–10 male mice (**A**,**B**,**E**,**F**) and 8–10 female mice (**C**,**D**,**G**,**H**) per group. Two-way ANOVA with repeated measures (genotype (Gen), time; * *p* < 0.05, **** *p* < 0.0001) was used for summary statistics; data points with differing superscript letters (a,b) differ from each other by *p* = 0.05 or less after Tukey’s post-hoc test.

**Figure 3:**
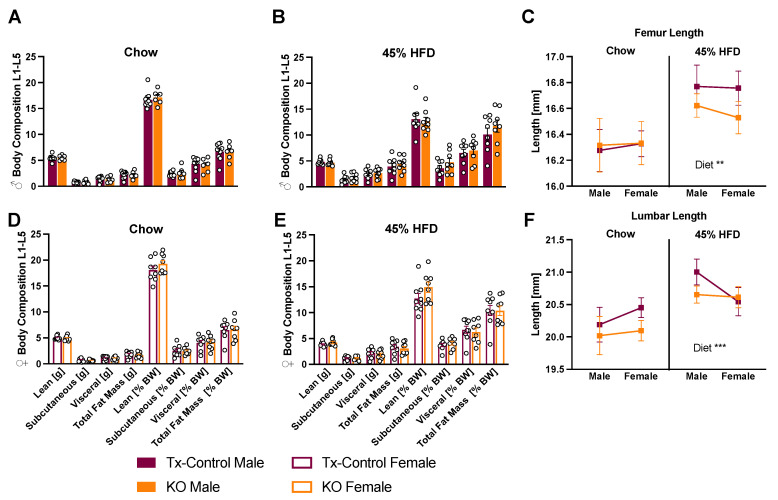
CTR depletion in microglia does not alter body composition or skeletal growth. Lean and fat mass were quantified post mortem in knockout mice and tamoxifen controls. Subcutaneous and visceral fat were quantified separately, but no significant difference for males (**A**,**B**) or females (**D**,**E**) was detected. 45% HFD led to more body fat (**B**,**E**) and to an increase in skeletal growth independent of genotype or sex (**C**,**F**); Skeletal length showed a diet effect after mixed-effects analysis. Values are displayed as mean ± SEM; n = 6–10 per group. Summary statistics after three-way ANOVA (Diet, sex, and genotype) detected a diet effects (**C**,**F**) *** *p* < 0.01 ** *p* < 0.001.

Two cohorts of mice fed ad libitum chow or 45% high fat diet (HFD) were weighed weekly for 10–11 weeks. Male KO mice showed significantly lower weight gain compared to oil control groups regardless of diet (Figure 2B,F). Female mice showed no intergroup differences on chow diet (Figure 2C,D), but oil-cre control mice gained more weight on HFD compared to KO mice (Figure 2H). Even though male and female CTR KO chow-fed mice had the lowest body weight gain of all four groups, the difference to tamoxifen controls (Tx-control) was non-significant. Body composition analysis showed no difference in lean or fat mass between KO and Tx-controls (Figure 3A,B,D,E), while diet had a significant effect on body fat and skeletal growth (Figure 3C–F).

### 2.3. Amylin and/or Leptin Injections Marginally Affect Food Intake as a Function of Genotype

**Figure 4:**
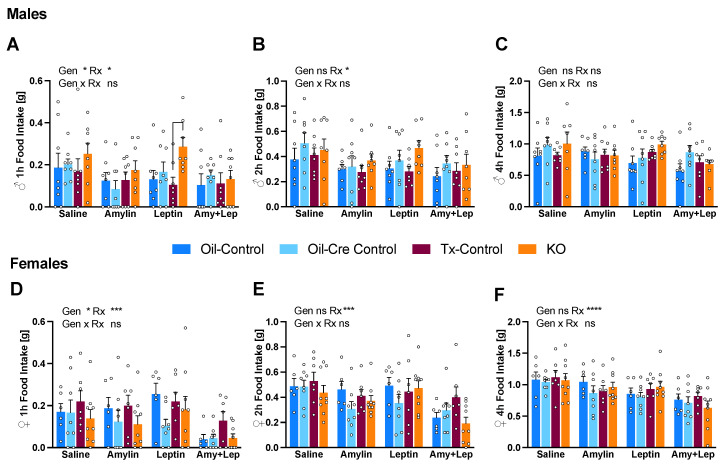
CTR depletion in microglia has no clear effect on food intake after amylin and leptin acute injection**.** Chow diet-fed mice (cohort 1) were fasted for 12 h during light phase and injected with a single dose of amylin (i.p.; 50 µg/kg), leptin (i.p.; 5 mg/kg), or a combination of both in a crossover study design with three days of washout between injections. Food intake 1 h, 2 h and 4 h post-injection is displayed for male (**A**–**C**) and female (**D**–**F**) mice. Data are displayed as mean ± SEM; n = 8–10 male mice and 6–9 female mice per group. Summary statistics after two-way ANOVA (genotype = Gen; treatment = Rx; * *p* < 0.05, *** *p* < 0.001, **** *p* < 0.0001) detected treatment effects (**A**,**B**,**D**–**F**) and genotype effects (**A**,**D**) but only one significant intergroup difference was found after Tukey’s post-hoc test (1 h food intake after leptin treatment, Tx-control vs. KO, (**A**)).

To determine the effect of the CTR depletion in microglia on the anorectic response to leptin and amylin, chow diet fed mice were injected with 50 µg/kg of amylin and/or 5 mg/kg of leptin at dark onset after a 12 h fast. In male mice, we observed a significant treatment effect at 1 h and 2 h for cumulative food intake regardless of the peptide (Figure 4A,B). The anorectic response to leptin was decreased at 1 h in KO mice with leptin-treated Tx-controls eating 68% less than KO (Figure 4A). In female mice, there was a consistent treatment effect at all time points but no difference between control and KO mice was noticed in the anorectic response to the different peptides (Figure 4D–F). A genotype effect was observed at 1 h but no intergroup difference was detected (Figure 4D). Amylin’s anorectic effect was similar across all groups.

### 2.4. CTR Microglial KO Has No Effect on Energy Expenditure but Decreases RER of HFD-Fed Male Mice

**Figure 5:**
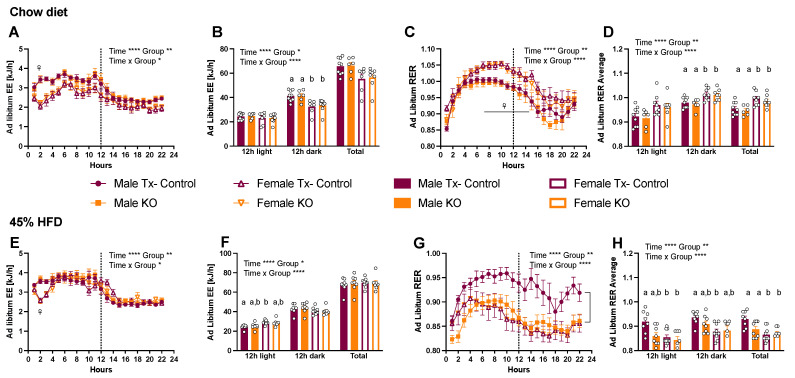
CTR depletion in microglia decreases respiratory exchange ratio (RER) in HFD male KO mice but has no effect on energy expenditure (EE). Ad libitum EE and RER measurements in Tx-controls and KO male and female mice fed chow (cohort 2; (**A**–**D**)) and 45% HFD (cohort 3; (**E**–**H**)). Hourly values are displayed as mean ± SEM n = 6−10 per group. Data were analyzed using a two-way ANOVA with repeated measures for genotype (Gen) and time * *p* < 0.05, ** *p* < 0.01, **** *p* < 0.0001. ^☥^ *p* < 0.05 male vs. female. Data points with differing superscript letters (a, b) differ from each other by *p* = 0.05 or less after Tukey’s post-hoc test. The shaded grey area indicates the dark phase.

Energy expenditure was similar between Tx-control and KO mice when fed chow or HFD (Figure 5A,B,E,F). However, chow-fed female mice had a lower EE compared to male mice due to their lower body weight (Figure 5B). Chow-fed female mice also displayed a higher RER throughout the dark and light phase compared to male mice (Figure 5C,D). Interestingly, RER of HFD-fed male KO mice was lower in the light phase compared to Tx-control mice, suggesting a preference for fat as a predominant fuel (Figure 5G,H).

### 2.5. CTR Microglial KO Does Not Alter Glucose Homeostasis

**Figure 6:**
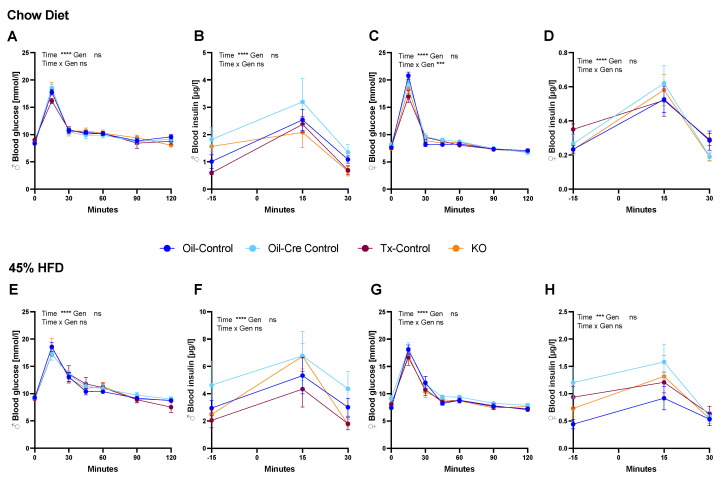
CTR depletion in microglia does not impair glycemic control. Chow or 45% HFD-fed mice received an oral gavage of 2 g/kg glucose after a 2 h fast. A first OGTT was performed for blood glucose measurements (**A**,**C**,**E**,**G**). Two weeks later, a second OGTT was performed for insulin measurements where tongue blood was collected after a brief isoflurane anesthesia (**B**,**D**,**F**,**H**). No significant genotype effects were detected among the groups. Baseline blood glucose and insulin levels were similar across sexes and diet, whereas HFD-fed mice were hyperinsulinemic (**F**,**H**) and female mice generally had lower levels of insulin (**D**) than males (**B**), as noticed by the scale differences on insulin graphs (**B**,**D**,**F**,**H**). Data are shown as mean ± SEM; n = 6–10 per group; statistical analysis two-way ANOVA with repeated measure for genotype and time; *** *p* < 0.001, **** *p* < 0.0001.

Oral glucose tolerance tests (OGTT) were performed twice on chow- and HFD-fed mice. Glucose tolerance was similar among all four groups on chow and HFD (Figure 6A–C,E–G). HFD only led to a moderate delay in glucose clearance at 30 min after gavage (Figure 6E,G). Peak insulin levels were 2.3-fold higher in HFD-fed mice compared to chow and 4.5-fold higher in male compared to female mice, but no significant differences between genotypes were observed (Figure 6B,D,F,H).

### 2.6. CTR Microglial KO Impairs Amylin-Induced pSTAT3 in the ARC but Not the Anorectic Response to Leptin

**Figure 7:**
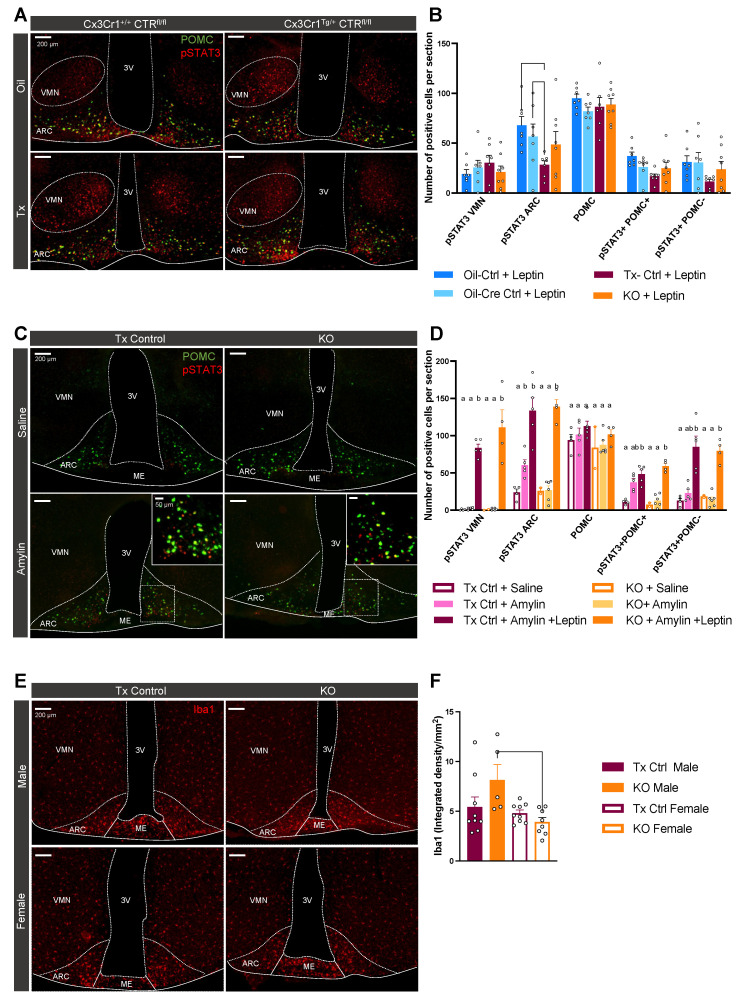
Amylin-induced pSTAT3 is decreased in the ARC of KO mice but leptin- and amylin + leptin-induced pSTAT3 is similar to Tx-controls. Chow-fed mice (cohort 1, n = 7–8/group) were injected with leptin (5 mg/kg i.p.) 45 min before sacrifice. Brain sections were double-stained for pSTAT3 (red) and POMC (green) (**A**) and quantified (**B**). A second cohort of chow-fed mice (cohort 2, n = 4–5/group) were injected with amylin (50 ug/kg i.p.) or amylin + leptin (5 mg/kg i.p.) 45 min before sacrifice. Brain sections were double-stained for pSTAT3 (red) and POMC (green) (**C**) and quantified (**D**). HFD-fed mice (cohort 3, n = 5–9/group) brain sections were stained for Iba1 (red) (**E**) and quantified (**F**). Data are displayed as mean ± SEM; data points with differing superscript letters (a,b) differ from each other by *p* = 0.05 or less after Tukey’s post hoc test for multiple comparisons. VMN: Ventromedial hypothalamic nucleus, ARC: Arcuate nucleus, 3V: Third ventricle, ME: median eminence.

To test the neuronal response to amylin and leptin, pSTAT3-positive cells were quantified in the ARC and VMN. Positive cells in the ARC were then split into POMC+ and POMC− populations. Leptin-induced pSTAT3 signaling was not altered in KO mice but Tx-controls showed a significantly lower number of pSTAT3+ cells in the ARC; this reduction was observed in POMC+ and POMC− neurons (Figure 7A,B). No significant genotype differences were observed for pSTAT3 expression in the VMN.

Notably, amylin failed to activate pSTAT3 signaling in KO mice and the number of pSTAT3 positive cells was decreased by 55% in the ARC compared to Tx-control mice (Figure 7C,D). This absence of response was mostly present in POMC+ neurons. This decrease in amylin-induced pSTAT3 was not translated at the behavioral level with amylin’s anorectic effect being similar across all groups (Figure 4A). As expected, no amylin-induced pSTAT3 was detected in the VMN (Figure 7C). When combined with leptin, a strong pSTAT3 response was detected but no difference between KO and Tx-control mice was observed.

Last, Iba1 density in the ARC was lower in female KO than in male KO but no difference with their respective controls was measured (Figure 7E,F).

## 3. Discussion

In this study, we aimed to probe an upstream mechanism for the amylin/leptin interaction in the mediobasal hypothalamus and specifically tested our working hypothesis of whether amylin acting via the CTR receptor on hypothalamic microglia mediates its leptin-sensitizing effect [6] and impacts the control of energy balance and glucose homeostasis in mice. We built this hypothesis based on our previous results showing that in rats, amylin treatment resulted in vitro in the release of IL-6 from hypothalamic explants and specifically from microglia, and that in vivo, the third ventricle infusion of IL-6 increased VMH leptin signaling [15]. Further, DIO rats primary ARC neurons treated for 5 days with IL-6 improved their response to leptin [23]. In addition to our own studies, the presence of functional amylin receptors on microglia has been demonstrated in studies on amyloid-induced neuroinflammation [27]. Hence, using a microglial specific KO of the main subunit of the amylin receptor, we investigated in vivo a possible direct activation of microglia by amylin to improve ARC neuron leptin signaling.

Our results show that the selective and temporal depletion of CTR in microglia did not seem to significantly impair energy homeostasis in mice. While we would have expected body weight to be increased in KO mice due to the lack of endogenous amylin signaling to enhance neuronal leptin signaling, male KO mice even showed a paradoxical weight loss compared to WT controls, but this effect can be partially attributed to the Tx treatment. Oral glucose tolerance tests and energy expenditure were not altered by the microglial CTR depletion either.

To further understand the underlying contribution of CTR microglial signaling on leptin sensitivity, we probed whether microglial amylin signaling interferes with the pSTAT3 neuronal response in the ARC. We found that leptin- and leptin/amylin-induced pSTAT3 in KO mice was similar to control groups. Previous experiments in rats showed that a non-cell specific short hairpin RNA-mediated knockdown of CTR in the VMH reduces leptin binding and consequently leptin-induced pSTAT3, leading to higher fat mass and to an increase of insulin release during glucose tolerance tests [7]. Exogenous, repeated amylin treatments in rodent models demonstrated that conversely, the increase in hypothalamic pSTAT3 signaling correlates with better leptin responsiveness [22] and results in weight loss [20]. In this study, we did not observe any correlation between pSTAT3 activation and the feeding response. Indeed, although leptin‘s anorectic response was blunted in the KO mice at 1 h post-injection, leptin-induced pSTAT3 was similar to controls.

We however noticed that amylin-induced pSTAT3 in the ARC was decreased in KO mice compared to Tx-controls even though the anorectic response to amylin was similar to control groups. Turek and colleagues also determined a pSTAT3 response after amylin application [16]. Since we have shown here that this effect is absent when CTR is depleted from microglia, it may be the result from an indirect effect through microglial IL-6 secretion [15] and its gp130 receptor on adjacent neurons [28]. The physiological consequences of amylin-induced pSTAT3 in adult animals remains unknown. Indeed, our KO mouse model did not display major metabolic alterations resulting from this defective signaling pathway. Nevertheless, we observed that HFD-fed male KO mice presented a lower RER, suggesting a preference for fat as a fuel compared to Tx-controls, but neither body composition nor body weight were affected. This is contrast to what we previously found in RAMP1/3 whole body KO, which displayed a preference for carbohydrate utilization under a HFD regimen [12]. In our previous studies, we determined that amylin directly activated pERK in POMC neurons but not in NPY neurons [11]. Here, we also show that the KO of the CTR in microglia affects amylin-induced pSTAT3 in POMC neurons while our initially hypothesized that amylin-induced IL-6 microglial signaling would rather affect pSTAT3 signaling in AgRP neurons.

Amylin exerts a strong effect on brain development during the early postnatal phase, in particular to promote axonal outgrowth of POMC and AgRP neurons from the ARC to the PVN [6,7,15,22]. Here, we used a Tx-inducible Cre model to exclude developmental effects and to focus on the role of amylin signaling during adulthood. We previously tested the response to the temporal loss of CTR in POMC neurons and found that even without developmental effects, the loss of amylin signaling lead to decreased energy expenditure, decreased locomotor activity and higher body fat in male mice independent of leptin signaling [11].

Taking the results from this study into account, endogenous amylin does not seem to act directly on microglia in a way that would alter the central leptin response and explain the amylin/leptin synergistic effect on body weight [21]. Otherwise, we would have expected to see a clear difference between control and KO mice in the leptin-induced pSTAT3 response in the ARC/VMN, on body weight and body fat [7]. Nevertheless, in vitro experiments have shown that IL-6 as a downstream mediator of amylin plays an important role in mediating these effects in the VMH. This leaves the question of whether a different upstream pathway leads to IL-6 release by microglia, or if the observed leptin-sensitizing effects are only present at pharmacological concentrations of amylin after exogenous application rather than endogenous amylin release. It is also possible that IL-6 is released by various cell types as it has been demonstrated in the context of acute hypoxia where the CNS with pharmacologically suppressed microglia is still capable of producing IL-6 [29]. To this end, additional experiments would need to evaluate whether IL-6 release in Cx3Cr1^CTR^ KO mice is diminished at all, and how these KO mice respond to sub-chronic amylin treatment.

Furthermore, we may hypothesize that the absence of effect on whole body energy homeostasis may be the result of a whole-brain CTR-microglia depletion rather than targeted depletion; indeed, CTR are present in many brain nuclei [30] and the whole brain depletion may have altered other signaling pathways that may have compensated for any effect we could have observed by only depleting CTR in ARC/VMN brain nuclei.

Finally, most of previous experiments were performed in rats and we may hypothesize that mice and rat amylin signaling may differ at least in part. Indeed, recently published data pointed out some species difference. For example, we observed that while sub-chronic amylin exerts a body lowering effect in both species, salmon calcitonin, an amylin agonist, does not have the same effect in mice while it does so in rats [13]. We are currently investigating the mechanism leading to this difference. Using newly available methods, such as single cell mRNA sequencing, it would be of great interest to determine whether some genes are more or less expressed in certain cell types in response to an amylin stimulus in the mediobasal hypothalamus of rats versus mice, similar to what has been performed in the dorsal vagal complex [31].

### Effects of Diet, Sex and Tamoxifen

Aside from the main question of microglial CTR signaling, we made some interesting observations thanks to robust cohorts representing both sexes. Tx is known to influence energy expenditure in experimental mice [32] and we observed a similar impact in POMC-Cre^ERT2^ mice [11]. In this study, we reduced the dose to 75 µg/g (50% less than in our previous study), but interestingly, male mice were more sensitive to Tx than females and gained less body weight on HFD after Tx treatment.

Following HFD and Tx treatment at ≥56 day of age, we could also still see a considerable effect of a high caloric diet on skeletal growth in the remaining growth phase. Ten weeks of HFD were enough to increase body fat to a point where mice showed clear signs of insulin intolerance. The measurement of insulin levels in a glucose tolerance test seems to be more suited to detect early pre-diabetic states in experimental mice without using labor-intensive clamp techniques [33]. Notably, there was an approximately 4.5-fold sex difference in insulin levels with females having lower values than males. In C57BL/6J mice, a clear sex difference in streptozotocin-induced diabetes has been described with females being more resilient to the treatment [34]. In human studies, women are generally more insulin sensitive than men, a difference which seems to diminish in the development of type 2 diabetes [35]. In this study, the relative increase of insulin under HFD was comparable between sexes, but the measured concentration was strongly diverging.

In conclusion, amylin action on microglial cells promotes pSTAT3 neuronal activation but is not sufficient to affect leptin signaling and to impact energy homeostasis. Thus, the underlying mechanism by which amylin/leptin synergistically mediate anorexia and body weight loss remains to be determined.

## 4. Materials and Methods

### 4.1. Animal Husbandry and Diet

Mice were kept in temperature-controlled rooms (21 ± 2 °C) with reversed 12 h light cycle (lights off at 10:00 AM). Three successive cohorts of 66–69 mice were generated from the same breeding pairs and randomly distributed into age- and sex-matched groups. At a minimum age of 56 days, mice were treated with tamoxifen (Tx) or corn oil. Cohorts 1 and 2 were fed a standard chow diet (No: 3430, energy content 3.15 kcal/g; Provimi Kliba AG, Kaiseraugst, Switzerland) and cohort 3 was fed 45% high fat diet (No: D12451, energy content 4.7 kcal/g; Research Diets, New Brunswick, NJ, USA) ad libitum (Figure 1B). Mice were kept in an enriched environment and group housed, except for experiments in indirect calorimetry cages and BioDAQ food intake measurements. The Veterinary Office of the Canton Zurich, Switzerland approved all animal procedures under the license 102/2018.

### 4.2. Transgenic Mice

Cx3Cr1-creER^T2^ (*B6.129P2(C)-Cx3cr1tm2.1(cre/ERT2)Jung/J;* MGI: 5467985) [36] were acquired from Jackson (Stock No: 020940) and genotyped according to Jackson instructions. CTR^fl/fl^ *(Calcr < tm1(fl)>;* MGI:5751436) were bred in our facility with frozen sperm kindly provided by Dr. Jean-Pierre David and Dr Thorsten Schinke, University Medical Center Hamburg [37,38]. We then crossed both genotypes to generate Cx3Cr1^+/+^ - CTR^fl/fl^ and CxCr1^Tg/+^ - CTR^fl/fl^ mice used for breeding and experiments (Figure 1A). Ai14 reporter mice *(B6.Cg-Gt(ROSA)26Sor < tm14(CAG-tdTomato)Hze>/J;* Stock No: 007914) [24] were bred with CxCr1^Tg/+^ mice to confirm specific Cre activity after tamoxifen treatment through removal of a floxed STOP cassette that results in the expression of tdTomato (Figure 1C,D).

### 4.3. Tamoxifen Induction

Tamoxifen (No: T5648; Sigma, Merck KGaA, Darmstadt, Germany) was slowly dissolved in ethanol and corn oil (No: C8267; Sigma) with gentle agitation and heating and then stored at −20 °C in aliquots until the day of use. Mice were injected at ≥56 day of age with 75 µg/g once daily intraperitoneally (i.p.) for 5 days, controls received an equal volume (1.5 mL/kg) of corn oil.

### 4.4. Food Intake Measurements after Amylin/Leptin Treatment

Mice were single housed in BioDAQ cages (Research Diets, New Brunswick, NJ, USA) and acclimated for one week. After a 12 h fast during the light phase, mice were injected with amylin (50 µg/kg i.p.; No: H9475; Bachem, Bubendorf, Switzerland), leptin (5 mg/kg i.p.; No: 450-31; Peprotech, London, UK), saline (NaCl 0.9% i.p.) or leptin + amylin combined in a crossover design and food was returned at dark onset. Food intake was then constantly monitored for 24 h after injection with intake after saline injections obtained as individual baselines. Meal pattern criteria were set at an inter-meal-interval (IMI) of 600 s and a minimal meal size of 0.02 g [39].

### 4.5. Glucose Tolerance Tests

After brief 2 h fasting before dark onset, mice were gavaged with a glucose solution (2 g/kg) at lights off using an intra-gastric gavage needle. Blood glucose was measured from tail tip sampling (ContourXT, Bayer, Germany) just before gavage and at 15, 30, 45, 60, 90, and 120 min after gavage. Blood insulin levels were measured 15 min prior to and 15, 30 min after gavage two weeks later with tongue blood (≤100 µL per sample) taken after a brief isoflurane anesthesia. Blood was collected in EDTA tubes on ice and centrifuged immediately at 4 °C. Plasma was stored at −80 °C until analysis with an ELISA kit (No: 10-1247-01; Mercodia, Uppsala, Sweden).

### 4.6. Indirect Calorimetry

Mice were single housed in a 16-cage TSE PhenoMaster open-circuit indirect calorimetry system for quantification of O_2_ consumption and CO_2_ production with an air flow rate of 0.41 L/min (TSE Systems; Bad Homburg, Germany). Following one week of adaptation, baseline metabolic activity and food/water consumption was measured for 24 h after fasting for 12 h during light phase, with sampling every 20 min. Energy expenditure (EE) and respiratory exchange ratio (RER) were calculated based on equations from Weir [40].

### 4.7. Measurement of Body Composition

CT scans of mouse carcasses were acquired with a Quantum GX micro-CT (PerkinElmer, Waltham, USA). X-ray settings were at 50 kV with 160 µA current for ideal soft tissue resolution. The full body scan was cropped to the length of the lumbar spine, femur, fat and lean mass was quantified as a representative portion of the whole body [41] with Analyze 12.0 software (AnalyzeDirect, Overland Park, KS, USA).

### 4.8. Mouse Perfusion

In a terminal experiment, mice were randomly injected with leptin (5 mg/kg i.p.), amylin (50 µg/kg i.p.), both or saline after a 12 h fast in the light phase. Forty-five minutes later, mice were injected with pentobarbital (100 mg/kg, Kantonsapotheke Zurich, Switzerland). After reaching deep anesthesia, mice received cardiac perfusion for 1.5 min with 0.1 M phosphate buffer (PB) followed by 2.5 min of 2% paraformaldehyde in 0.1 M PB (PB-PFA, pH 7.4). Brains were postfixed for 4 h in 2% PB-PFA and cryoprotected in 20% sucrose in 0.02 M potassium phosphate buffer saline (KPBS) for 24 h [22]. Cryoprotected brains were subsequently frozen in hexane on dry ice and stored at −80 °C. Frozen brains were cut in 25 µm slices in series of 4 on a cryostat (CM3050 S, Leica Biosystems, Germany) and mounted on Superfrost Plus slides (Thermo Fisher Scientific, Reinach, Switzerland) and stored in cryoprotectant (50% 0.02 M KPBS, 30% ethylene glycol, 20% glycerol) at −20 °C.

### 4.9. Immunohistochemistry (IHC)

**POMC-pSTAT3 double staining:** IHC was performed as previously described [22]. In brief, brain sections underwent antigen retrieval in NaOH and H_2_O_2_ followed by glycine and sodium dodecyl sulfate. After blocking for 2 h in 4% normal goat serum (NGS), 0.4% triton X-100 and 1% bovine serum albumin (BSA) in KPBS, sections were then incubated in rabbit anti-pSTAT3 (1:1000, No: 9145, cell signaling) for 48 h at 4 °C followed by Cy3 goat anti-rabbit (1:100, No: 111-165-144, Jackson ImmunoResearch Europe) for 2 h at room temperature. The blocking and staining steps were then repeated with rabbit anti-POMC (1:1000, No: H029-30, Phoenix Europe, Karlsruhe, Germany) primary antibody and AF488 donkey anti-rabbit secondary antibody (1:100, No: 711-545-152, Jackson). Sections were then counterstained with DAPI and cover slipped with hard-set Vectashield (No: H-1400-10, Vector labs).

**Iba1 Staining:** Sections were blocked in 0.3% Triton, 1% BSA, 2% NGS in PBS before incubation with primary antibody rabbit anti-Iba1 (1:1000; #019-19741, Wako Chemicals, USA) for 48 h at 4 °C followed by AF647 or AF488 goat anti-rabbit (1:100, No: 111-605-144, No: 111-545-144, Jackson) for 2 h at RT. Sections were then counterstained with DAPI and cover-slipped with hard set Vectashield.

### 4.10. Basescope In Situ Hybridization

Frozen brains were cut in 16 µm slices in series of 6 on a cryostat (CM3050S, Leica Biosystems, Germany) and mounted on Superfrost Plus slides (Thermo Fisher Scientific, Reinach, Switzerland) and stored at −80 °C for up to 3 weeks until processing.

A 3 ZZ Basecope probe was custom designed to target the mRNA sequence of the *calcr* gene between exon 6 and 7 (NCBI gene ID: 12311; channel 1: green stain), which are removed during the Cre-Lox process [37]. Sections were co-stained with *Aif1* (BA-Mm-Aif1-3zz-st-Mus musculus allograft inflammatory factor 1 corresponding to the *Iba1* gene; channel 2: red stain) (No: 891671; Bio-Techne, Zug, Switzerland). A duplex Basecope assay was run following the manufacturer’s instruction (No: 322982; Bio-Techne, Zug, Swizerland) and after a 12-step amplification and counterstaining with Gill’s hematoxylin 1 (No: GHS116; Sigma, Merck KGaA, Darmstadt, Germany), slides were dehydrated and coverslipped with Vectamount (No: H-5000-60; Vector Labs). Validation of the stain was confirmed with positive and negative controls. Slides were then visualized with the Axio Imager 2 microscope (Zeiss Germany) at 20× and 40×. Localization of *Calcr* and *Aif1* was then performed.

### 4.11. Imaging and Quantitative Analysis

Stained sections were imaged with an Axio Imager 2 microscope (Zeiss Germany) on a 10× objective and three sections of the hypothalamus at the level of the ARC per animal were quantified by an experimentally blinded observer. A 20 µm Z-stack consisting of 5 stacked images was summarized and analyzed using Fiji open-source software [42] by defining fixed thresholds for each channel and converting them to masks. Particles were counted at a size ≥20 square pixels for each channel and double-labeled particles were defined as mask overlaps of ≥10 square pixels.

### 4.12. Quantification and Statistical Analysis

Data are reported as mean ± standard error of the mean (SEM). Statistical analyses of physiological data were performed with Prism software (version 8 GraphPad Prism, La Jolla, CA, USA). For all statistical tests, a *p*-value less than 0.05 was considered significant. Information on replicates and significance is reported in the figure legends.

## Data Availability

Further information and requests for resources and reagents should be directed to and will be fulfilled by the Lead Contact Christelle Le Foll (christelle.lefoll@uzh.ch). Mouse lines generated by this study will be available upon request.
